# Immunomodulatory effects of *Rhipicephalus haemaphysaloides* serpin RHS2 on host immune responses

**DOI:** 10.1186/s13071-019-3607-4

**Published:** 2019-07-11

**Authors:** Zhengmao Xu, Zhibing Lin, Nana Wei, Qing Di, Jie Cao, Yongzhi Zhou, Haiyan Gong, Houshuang Zhang, Jinlin Zhou

**Affiliations:** Key Laboratory of Animal Parasitology of Ministry of Agriculture, Shanghai Veterinary Research Institute, Chinese Academy of Agricultural Sciences, Shanghai, China

**Keywords:** *Rhipicephalus haemaphysaloides*, Tick, Serpin, BMDC, Immmunomodulatory

## Abstract

**Background:**

*Rhipicephalus haemaphysaloides* is a widespread tick species in China and other South East Asian countries, where it is the vector of many pathogens. The objective of this study was to study the role of serpin (serine protease inhibitor) during the tick-host interaction.

**Methods:**

The differentiation of bone marrow-derived dendritic cells (BMDC) was induced *in vitro*, and the effect of RHS2 on the maturation of DCs was evaluated. The effects of RHS2 on T cell activation and cytotoxic T lymphocytes’ (CTLs) activity were analyzed by flow cytometry. Antibody subtypes after immunization of mice with RHS2 and OVA were determined.

**Results:**

RHS2 can inhibit the differentiation of bone marrow-derived cells into DCs and promote their differentiation into macrophages. RHS2 can inhibit the maturation of DCs and the expression of CD80, CD86 and MHCII. The number of CD3^+^CD4^+^ and CD3^+^CD8^+^ T cells secreting IFN-γ, IL-2 and TNF-α was decreased, and the number of CD3^+^CD4^+^ T cells secreting IL-4 was increased, indicating that RHS2 can inhibit the activation of CD4 T cells and CD8 T cells, leading to inhibition of Th1 immune response. RHS2 inhibits the elimination of target cells by cytotoxic T lymphocytes. After immunization of mice with RHS2 and OVA, serum IgG2b was significantly reduced and IgM was increased.

**Conclusions:**

The results show that RHS2 has an inhibitory effect on the host immune response. Ticks have evolved various ways to circumvent adaptive immunity. Their serpin inhibits BMDC differentiation to reduce immune responses.

## Background

Ticks are hematophagous arachnid ectoparasites characterized by a complex developmental life-cycle. Following hatching, ticks have three development stages: larva, nymph and adult (male and female) [1]. Widely distributed throughout the world, ticks are known to parasitize mammals, including humans, birds, reptiles and even amphibians [2]. Taxonomically, ticks are divided into three families: Ixodidae, Argasidae and Nuttallidae. Ticks are second only to mosquitoes in importance as vectors of pathogens causing human and animal diseases [3]. In human and veterinary medicine, ticks are direct external parasites and also vectors of important pathogens. Ticks transmit tick-borne diseases (TBD), including parasitic, viral, bacterial and spirochete diseases [2, 4]. Ticks and TBD have been significant problems plaguing animal production in many countries [3, 5, 6]. The control of ticks and TBD relies on the use of acaricides. However, because of environmental pollution [7] and acaricide resistance [8–10], it is important to develop new and more sustainable control strategies.

Serine protease inhibitor (serpin) plays an important role in the regulation of endogenous protease balance [11], innate immune response [12], pathogen transmission [13, 14], food digestion [15, 16], blood-sucking [17–19] and egg production [18, 20]. It also affects host blood coagulation [11, 21–24] and immune response modulation [14, 25–27]. The inflammatory response also has an important effect in the host. Iris, a protein secreted by *Ixodes ricinus*, suppresses proliferation of T lymphocytes, induces a Th2 immune response by increasing IL-4 secretion, and regulates the innate immune response by inhibiting the production of typical Th1 cytokines (IL-2 and IFN-γ) [28]. *Ixodes ricinus* salivary serpin IRS2 inhibits acute inflammatory responses, swelling and the migration of neutrophils into the inflamed tissue [14, 29]. IRS2 also inhibits Th17 cell differentiation by downregulating the IL-6/STAT-3 signaling pathway [14]. IRS2 is a serpin inhibitor that targets cathepsin G and chymotrypsin. These two proteases are secreted after the activation of neutrophils (cathepsin G) and mast cells (chymotrypsin) and they are involved in various types of physiological processes of acute inflammatory reactions [29], especially the interactions between neutrophils and platelets [30]. IRS2 also inhibits thrombin-induced platelet aggregation and it may have multiple roles in inflammation and anticoagulation, particularly by regulating the activation of protease-activated receptors (PAR) [29]. The effects of serpin from other arthropods on innate immunity cells and their function in driving adaptive immune responses have been studied [31–34].

To avoid attack by the host immune system during blood-feeding, ticks secrete protein and non-protein molecules which have anticoagulant and immunomodulatory effects [35]. The surfaces of dendritic cells (DCs) have pattern recognition receptors (PRRs) that specifically recognize pathogen-associated molecular patterns (PAMPs). They also participate in the uptake of antigens, expressed *via* MHC class I (MHCI) and class II (MHCII) molecules. In antigen presentation they play a role in the initiation and regulation of immune responses [36]. After DCs take up antigens, they process them so that they become associated with the major histocompatibility complex (MHC) molecules and be presented on the cell surface [37]. DCs activate T lymphocytes as a result of antigen presentation, creating a unique link between innate and acquired immune responses [37]. Antigen processing by DCs primarily occurs through two major pathways: an exogenous (endosomal) pathway and an endogenous (proteasomal) pathway, CD4 and CD8 T cells respond to peptide antigen displayed on MHC class II and MHC class I molecules [36].

During tick-feeding, ticks digest host cells. In the tick gut epithelium, nutrient endocytosis and lysosome maturation facilitate intracellular digestion [38, 39]. The luminal surface of the midgut can be accessed by the host immune effectors and the blood components ingested during blood-feeding [40]. It is unclear how the midgut cells inhibit multiple proteases and effector molecules in the host blood to maintain homeostasis and protect the midgut cells from damage. Most inhibitors have been found in the salivary glands but relatively few have been identified in the midgut [41]. *Rhipicephalus haemaphysaloides* serpin 2 (RHS2) is only expressed in the midgut and is able to significantly inhibit chymotrypsin activity [42]. RNA interference studies have shown a significant decrease in tick attachment and engorgement rates. These results reveal that RHS2 is involved in successful tick blood-feeding [42].

It is important to determine the role of tick serpin on host immune effector molecules and its role in blood digestion. In this study, we studied the effects of RHS2 on host immune regulation.

## Methods

### Animals and ticks

Six-eight-week-old female C57BL/6 mice were purchased from SLAC Laboratory Animal Co., Ltd. (Shanghai, China). They were maintained following the approved guidelines from the Animal Care and Use Committee of the Shanghai Veterinary Research Institute (approval number SHVRI-MO-2018010020). *Rhipicephalus haemaphysaloides* were maintained under standard conditions in the animal facilities of the Shanghai Veterinary Research Institute, Chinese Academy of Agricultural Sciences (Shanghai, China).

### Reagents and chemicals

Lipopolysaccharide (LPS) (*Escherichia coli*, *E. coli* 055:B5) and PMA [phorbol 12-myristate 13-acetate ≥ 99% (TLC), film or powder] were purchased from Sigma-Aldrich (St. Louis, MO, USA). Ionomycin (Free Base) was purchased from MKbio (Shanghai, China). Recombinant mouse GM-CSF (granulocyte-macrophage colony stimulating factor), IL-4 (interleukin 4) and IL-2 (interleukin 2) were obtained from Peprotech (Rocky Hill, NJ, USA). Cell staining was performed by using the following mAbs from BD Biosciences (Franklin Lakes, NJ, USA): anti-CD45 FITC (30-F11, #553079), anti-CD11b PerCP-Cy5.5 (M1/70, #550993), anti-CD11c APC (HL3, #550261), anti-F4/80 BV421 (T45-2342, #565411), anti-Ly-6G (Gr-1) PE (1A8, #551461), anti-CD80 PE (16-10A1, #553769), anti-MHC II BV421 (M5/114.15.2, #562564), anti-CD86 PE (GL1, #561963), anti-CD40 BV421 (3/23, #562846), anti-CD3 FITC (17A2, #561798), CD4 PerCP-Cy5.5 (RM4-5, #550954), anti-CD8a PerCP-Cy5.5 (53-6.7, #551162), anti-IL-2 PE (JES6-5H4, #554428), anti-TNFα BV421(MP6-XT22, #563387) and anti-IFNγ APC (XMG1.2, 554413). The isotype-matched mAbs for control staining were from BD Biosciences. Cytotoxicity LDH Assay Kit-WST was purchased from Dokindo (Tokyo, Japan).

### Expression and purification of RHS2

The recombinant plasmid expressing RHS2 (PGEX-6P-1-RHS2) was transformed into *E. coli* BL21 (DE3). Then, the transformants were induced with 1 M isopropyl-b-d-1-thiogalactopyranoside (IPTG) at 20 °C to express the protein for 20 h. After induction, the bacterial cells were harvested, ultrasonicated and centrifuged. The recombinant proteins were highly expressed and soluble. The supernatant was purified with a GST resin column (Novagen, Madison, WI, USA). After purification, the GST-tag recombinant proteins were PreScission Protease (Sigma, # SAE0045, USA) cleaved at 4 °C for 16 h. The cleaved proteins were examined by 12% sodium dodecyl sulphate-polyacrylamide gel electrophoresis (SDS-PAGE). The cleaved proteins were treated by Detoxi-Gel Endotoxin Removing Gel (Thermo Fisher Scientific, Waltham, MA, USA) to remove the endotoxin. Finally, the concentration of RHS2 was determined by a BCA protein assay to remove endotoxins.

### Bone marrow-derived dendritic cells (BMDC) culture

BMDC were generated as previously described with some minor modifications [43, 44]. Briefly, BM cells were removed from the femurs and tibias of C57BL/6 mice and the marrow was flushed with RPMI-1640 (Gibco, Carlsbad, CA, USA) using a syringe with a 0.45-mm needle. Clusters within the marrow suspension were disassociated by rapid pipetting, and cells were filtered through a 70-μm cell strainer. BM cells were cultured in 6-well culture plates, at a concentration of 1.5 × 10^6^ cells per well, in 1.5 ml of RPMI-1640 supplemented with heat-inactivated 10% fetal bovine serum (New Zealand FBS, Gibco), 100 U/ml of penicillin, 100 μg/ml of streptomycin, GM-CSF (40 ng/ml) and IL-4 (20 ng/ml). On day 3 of culturing, the same volume of the supplemented medium was added. On days 6 and 9, the supernatant was gently removed and replaced with the same volume of fresh medium. On day 9 of culturing ≈ 90% of the cells were CD11c^+^ DCs.

### Isolation of splenocytes and splenic lymphocytes

Female C57BL/6 mice (6–8-weeks-old, SPF) were killed by CO_2_ asphyxiation and their spleens were removed aseptically and placed into ice-cold phosphate-buffered saline (PBS, Gibco). Single cell suspensions were prepared by gentle dispersion of the cells and straining through a 70-μm nylon strainer. Clustered cells were removed by filtration through a 40-μm nylon strainer, and washed twice with PBS. Lymphocytes were separated using Ficoll–Hypaque gradients and were resuspended in RPMI 1640 medium (Gibco) containing 10% FBS.

### CD4 and CD8 T cell activation

Dendritic cells were cultured for 10 d in culturing in 6-well culture plates at a concentration of 1.5 × 10^6^ cells per well in 1.5 ml of RPMI-1640 supplemented with heat-inactivated 10% FBS, 100 U/ml of penicillin, 100 μg/ml of streptomycin, OVA-CD4 (KISQAVHAAHAEINEAG), or OVA-CD8 (SIINFEKL) peptide, in the presence or absence of RHS2 (20 μg/ml). BMDC were cultured at 37 °C in a humidified 5% CO_2_ incubator. After 36 h of incubation, 2 × 10^6^ of freshly isolated lymphocytes of culture medium was added to each well. The lymphocytes were incubated with DCs for 3 d before re-stimulation with PMA (20 ng/ml) and ionomycin (1 μM). After an additional 2 h, the cells were treated with the protein transport inhibitor brefeldin A (BD). The cells were then incubated for 4 h before staining was performed with anti-CD3 FITC (17A2), CD4 PerCP-Cy5.5 (RM4-5), anti-CD8a PerCP-Cy5.5 (53–6.7), anti-IL-2 PE (JES6-5H4), anti-TNF-α BV421 (MP6-XT22), anti-IL-4 PE (11B11) and anti-IFN-γ APC (XMG1.2) (BD Biosciences).

### Immunoblotting

Western blot analysis was performed as described previously [45]. In the present study, BM-derived DCs were collected on the 3rd and 6th days of culture. Cells were washed twice with cold PBS. They were then lysed in a modified RIPA buffer (Thermo Fisher Scientific), in the presence of phenylmethylsulfonyl fluoride (PMSF), protease and phosphatase inhibitor (Sangon Biotech Co., Ltd., Shanghai, China). They were then placed on ice for 10 min on a swirling plate to assure uniform spreading. The samples were then centrifuged at 12,000×g for 10 min to pellet the cell debris. Cell lysates from each sample were subjected to SDS-PAGE followed by the transfer of proteins to polyvinylidene fluoride (PVDF) membranes. Following blocking in Tris-buffered saline (TBS) containing 5% fat-free milk, the blots were incubated overnight with the antibodies against STAT-3 (124H6, #9139), phospho-STAT-3 (Tyr705, #9145), ERK (Thr202, #9102), phospho-ERK (Thr202, #9106), p38 (Thr180, #9212), phospho-p38 (Thr180, #9211) (all from Cell Signaling Technology Inc., Danvers, MA, USA) and β-actin (60008-1-Ig, Proteintech, Chicago, IL, USA). The signal was detected with an Enhanced Chemiluminescent Substrate Reagent Kit (NCM Biotech, Sunzhou, China) and it was visualized under a Tanon-5200 Chemiluminescent Imaging System (Tanon Science and Technology, Shanghai, China).

### Cytotoxic T lymphocyte (CTL) killing assay

Tumor cell line B16F10-OVA were the target cells of the assay. A subclone of cells was transfected with the cDNA of OVA. Cells were cultured in DMEM (Gibco) supplemented with 10% FBS, penicillin (100 U/ml) and streptomycin (100 µg/ml).

Mice were inoculated subcutaneously with 500 μg OVA-CD8 peptide plus incomplete Freund’s adjuvant to the flank once a week for 6 weeks. Lymphocytes (2 × 10^6^ cells/ml) were isolated from spleens 7 d after the third immunization. Spleens were harvested and homogenized into single cell suspensions, and a 70-μm cell strainer (FALCON, Durham, NC, USA) was used to remove debris. The cells were washed and resuspended in RPMI medium for counting. Cells were resuspended at 2 × 10^6^ cell/ml in RPMI complete medium, and they were incubated with 20 μg/ml OVA-CD8 peptide for 4 d at 37 °C.

B16F10-OVA were seeded (1 × 10^6^/well) in triplicate (100 μl/well) in 96-well plates cells. They were cultivated at 37 °C in a humid incubator for 2 h with 5% CO_2_. Then, 100 μl of lymphocytes (2 × 10^6^ cell/well) were added as effector (E) cells and cultivated with 20 µg/ml RHS2 for 2 to 4 h. Triton X-100 was added to the positive control group. The Cytotoxicity LDH Assay Kit-WST was used to measure the absorbance of all of the samples at 490–500 nm using a Spectramax microplate reader (Spectramax M5, Molecular Devices, San Jose, CA, USA).

### Mice immunization

Six-week-old female C57BL/6 mice were divided into three groups, each consisting of five mice. Animals were immunized subcutaneously with 50 μg of ovalbumin (OVA, Sigma-Aldrich) in the presence or absence of RHS2 (50 μg). PBS-treated animals were included as the controls. A boosting injection was given 14 d later. Serum was collected 10 d after the third immunization for antibody subtyping detection.

### Antibody subtype detection

Anti-OVA IgG, IgG1, IgG2a, IgG2b, IgG3, IgM and IgE antibodies in serum were detected by an indirect ELISA. Briefly, microtiter plate wells (Thermo Fisher Scientific) were coated with 100 μl OVA solution (1 μg/ml, in 50 mM carbonate-bicarbonate buffer, pH 9.6) for 14 h at 4 °C. The wells were washed three times with PBS containing 0.05% (v/v) Tween 20 (PBS/Tween, PBST), and then blocked with 5% skimmed milk powder (SMP) at 37 °C for 1 h. After three washings, 100 μl of diluted serum sample (1:100) or PBS as control was added to the triplicate wells. The plates were then incubated for 2 h at 37 °C, followed by washing 6 times. Aliquots of 100 μl of goat anti-mouse IgG horseradish peroxidase conjugate diluted 1:4000, goat anti-mouse IgG1, IgG2a, IgG2b, IgG3, IgM and IgE peroxidase conjugate 1:4000 with 5% SMP were added to each plate. The plates were further incubated for 1 h at 37 °C. After washing, the peroxidase activity was assayed as follows. A total of 100 μl of substrate solution (10 mg of 3, 3′, 5, 5′-tetramethylbenzidine and 37.5 μl of 30% H_2_O_2_ in 25 ml of 0.1 M citrate-phosphate buffer, pH 5.0) was added to each well. The plate was incubated for 10 min at 37 °C and the enzyme reaction was terminated by adding 50 μl/well of 2M H_2_SO_4_. The optical density (OD) was measured in an ELISA reader at 490 nm with a 595 nm reference. Data were expressed as the mean OD value of the samples minus the mean OD value of the control. When sets of serum samples were subjected to within- and between-group comparisons, ELISA assays were performed on the same day for all of the samples.

### Data analysis

GraphPad Prism 6 software (GraphPad Software Inc., San Diego, CA, USA) was employed for the statistical analysis. Comparisons between two groups were performed using the two-tailed unpaired Student’s t-test. When more than two groups were compared, one-way ANOVA with *post-hoc* Bonferroni’s test was used. *P* < 0.05 was considered to be significant and *P* < 0.01 was considered to be highly significant.

## Results

### Differentiation of BMDC

**Fig. 1:**
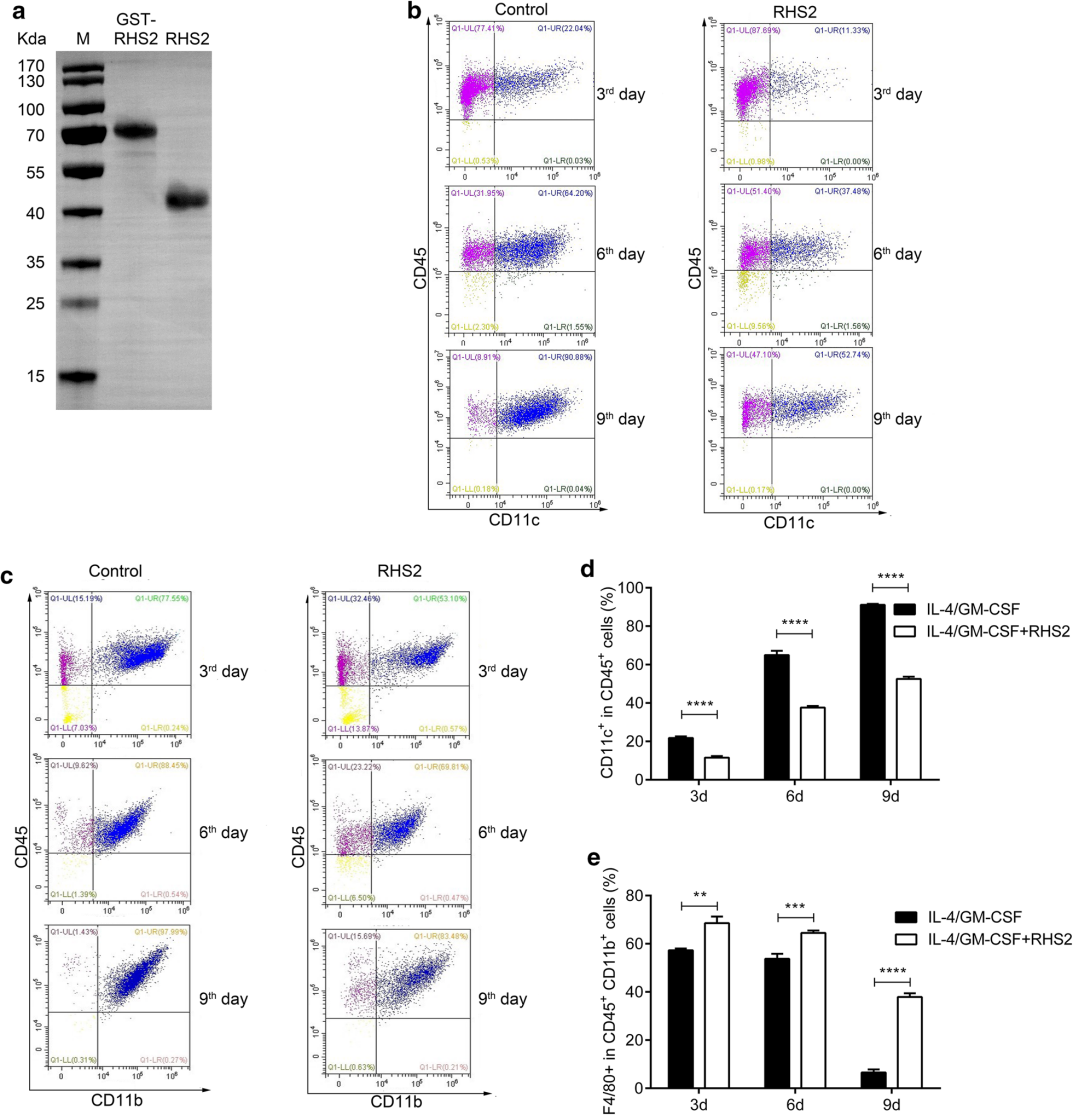
Differentiation of BMDC in the presence or absence of RHS2. **a** Recombinant RHS2 purified from a GST affinity column (GST-RHS2 and after cleavage of the GST tag, detected by Western blot). **b**, **c** Cultured cells were labeled with the designated monoclonal antibodies (mAbs) and analyzed by two-color flow cytometry. Results are shown as the mean percentage ± standard error of the mean (SEM) of CD45^+^ CD11c^+^ cells from duplicate wells. *P < 0.05, **P < 0.01, ***P < 0.001 and ****P < 0.0001 compared to cells cultured only with GM-CSF + IL-4 (control group). Data shown are representative of two experiments. **d** CD45^+^ CD11c^+^ and **e** CD45^+^ CD11b^+^ F4/80^+^, the results are shown as the mean percentage ± standard error of the mean (SEM)

Recombinant RHS2 was obtained using a prokaryotic expression system with a molecular weight of 42.7 kDa (Fig. 1a). The effect of tick serpin RHS2 on the differentiation of DCs from BM precursors was investigated by culturing BM-derived cells in the presence of GM-CSF, IL-4 and RHS2 (20 μg/ml, 2.4 μM) for 9 days. RHS2 significantly inhibited the differentiation of BM cells into CD45^+^ CD11c^+^ DCs (Fig. 1b) and CD45^+^CD11b^+^ cells (Fig. 1c). In order to verify whether the RHS2-inhibitory effect was directed against an early or a late phase of DCs differentiation, we collected the DCs on days 3, 6 and 9, and we determined the CD45^+^ CD11c^+^ cell population by two color flow cytometry. Our data showed that RHS2 significantly reduced DC (CD45^+^ CD11c^+^) differentiation on days 3, 6 and 9 of BM cell culture (day 3, IL-4/GM-CSF+RHS2: 11.54 ± 0.85% *vs* IL-4/GM-CSF: 21.78 ± 0.80%; t-test: *t*_(4)_ = 15.2, *P* = 0.00001; day 6, IL-4/GM-CSF+RHS2: 37.79 ± 0.87% *vs* IL-4/GM-CSF: 64.97 ± 2.22%; *t*_(4)_ = 19.87, *P* = 0.00001; day 9, IL-4/GM-CSF+RHS2: 52.56 ± 0.11% *vs* IL-4/GM-CSF: 91.12 ± 0.49%; *t*_(4)_ = 55.18, *P* = 0.00001; Fig. 1d, e). Results presented in Fig. 1 are representative of three independent experiments.

### Promotion of p38 and STAT3 phosphorylation

**Fig. 2:**
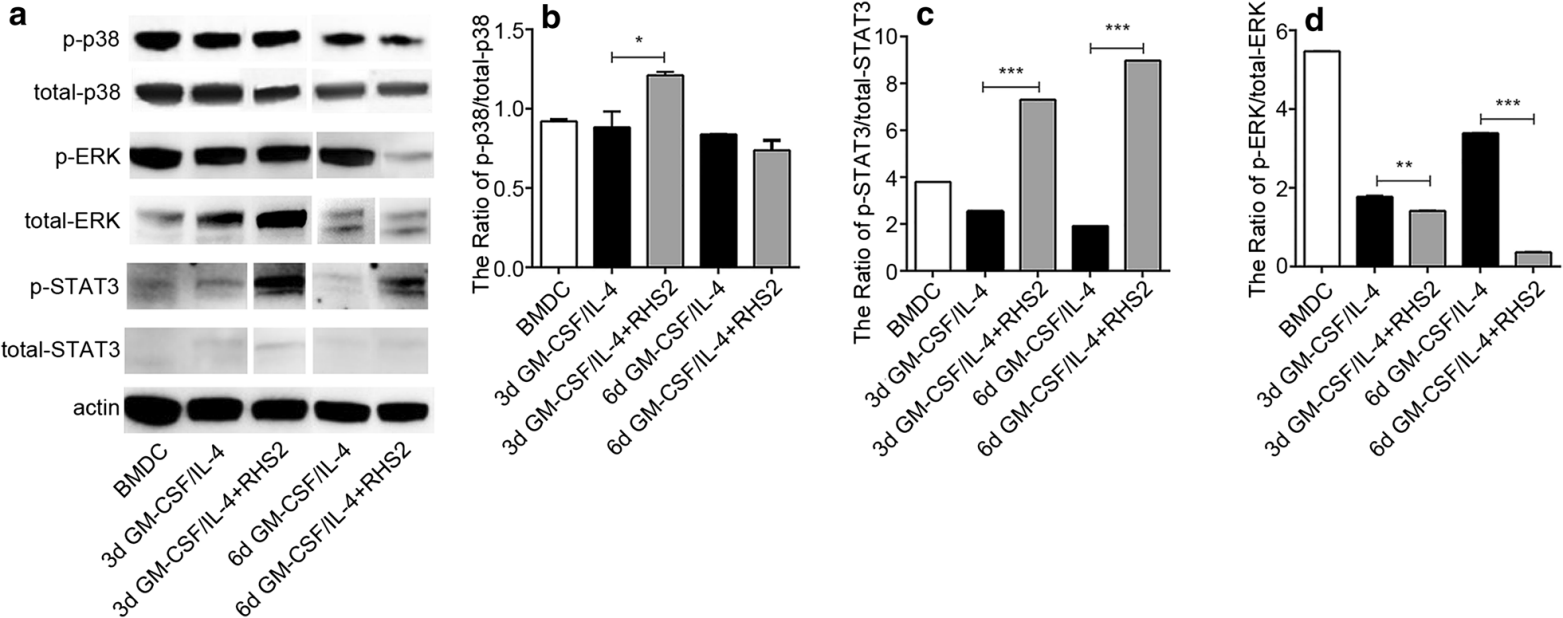
The effects of RHS2 on ERK, STAT3 and p38 phosphorylation. **a** Western blotting was performed to analyze the phosphorylation levels of ERK, STAT3 and p38 in DC differentiation, which was significantly higher in GM-CSF/IL-4 + RHS2 compared to GM-CSF/IL-4. **b**–**d** Gel-Pro analyzer 4 was used to detect gray value and the results were defined as normalized gray value of various experiments in reference to the control group. Each experiment was performed independently three times. *P < 0.05, **P < 0.01, ***P < 0.001

The JAK2/STAT3 signaling pathway plays an important role in cell proliferation, differentiation and apoptosis [46, 47]. The phosphorylated ERK1/2 can phosphorylate p90RSK and promote cell proliferation, survival and metastasis through targeted gene expression [48]. p38 MAPK are activated by cellular stress stimuli or cytokines, and they are associated with stress response, cytokine production, cell growth and apoptosis [49]. To further understand the mechanisms underlying the changes in DCs phenotype observed above, we investigated the involvement of ERK, STAT3 and p38 signaling effector by Western blot. We found that RHS2 induced upregulation of phosphorylated p38 (t-test: day 3, *t*_(2)_ = 4.533, *P* = 0.0454; day 6, *t*_(2)_ = 2.235, *P* = 0.155) and STAT3 (t-test: day 3, *t*_(2)_ = 6735, *P* = 0.0001; day 6, *t*_(2)_ = 9953, *P* = 0.0001; Fig. 2a–c), and downregulation of phosphorylated ERK (t-test: day 3, *t*_(2)_ = 17.35, *P* = 0.0033; day 6, *t*_(2)_ = 427.3, *P* = 0.0001; Fig. 2d).

### Maturation of BMDC

**Fig. 3:**
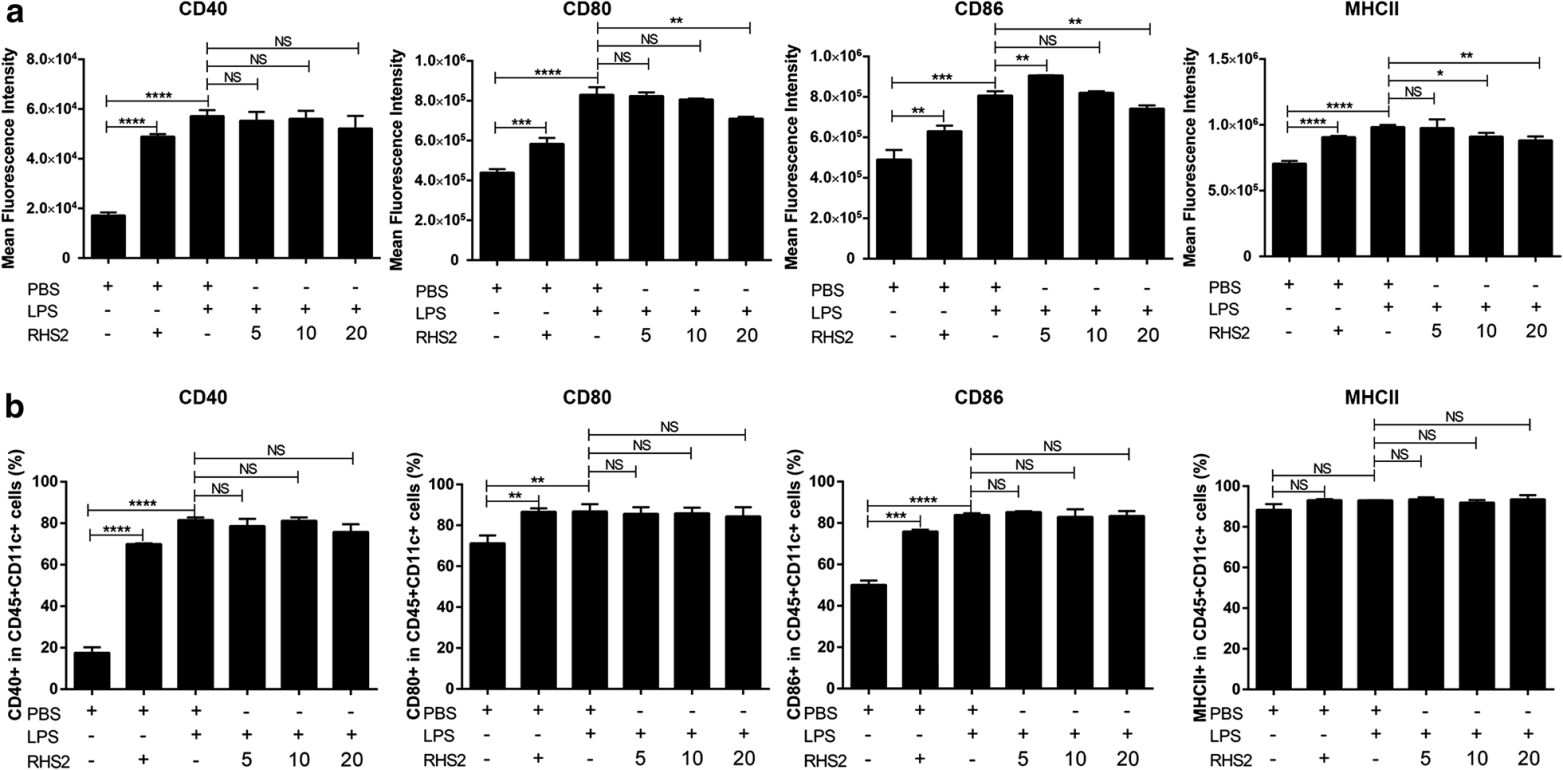
RHS2 inhibited the maturation of BMDC. **a** Expression of co-stimulatory molecules incubated with LPS or with RHS2 after 36 hours using flow cytometry, MFI levels are shown from three independent experiments. **b** The percentage of co-stimulatory and MHC-II molecule expression on CD45+ CD11c+ cells. Each experiment was performed independently three times. *P < 0.05, **P < 0.01, ***P < 0.001, ****P < 0.0001; bars represent the mean of three independent experiments ± SD

Activation and maturation of DCs are crucial for the initiation of cell-mediated immunity. The maturation of DCs is evaluated by studying the increase in the expression of CD40, CD80, CD86 and MHC-II proteins on the surface. BMs were cultured in the presence of GM-CSF and IL-4 for 9 days, and the differentiated immature DCs were harvested. The harvested DCs were stimulated by LPS for 36 h with or without RHS2. Then, the DCs were stained and analyzed for the expression of CD40, CD80, CD86 and MHC-II molecules by flow cytometry. The immature DCs stimulated with LPS showed significantly increased expression of CD40 (one-way ANOVA: *F*_(2, 6)_ = 502.3, *P* = 0.00001), CD80 (*F*_(2, 6)_ = 138, *P* = 0.00001), CD86 (*F*_(2, 6)_ = 64.99, *P* = 0.0001) and MHCII (*F*_(2, 6)_ = 230.9, *P* = 0.00001) compared with that of the control group. However, following LPS induction, we observed downregulation of CD80 (one-way ANOVA: *F*_(2, 6)_ = 16.08, *P* = 0.0028), CD86 (*F*_(2, 6)_ = 18.74, *P* = 0.0026) and MHCII (*F*_(2, 6)_ = 8.298, *P* = 0.0264; Fig. 3) and no change of CD40 (*F*_(2, 6)_ = 0.8821, *P* = 0.5015; Fig. 3). These results indicated the RHS2 inhibitory effect on BMDC maturation.

### IL-2, IFN-γ and TNF-α production in CD4^+^ and CD8^+^ T cells

**Fig. 4:**
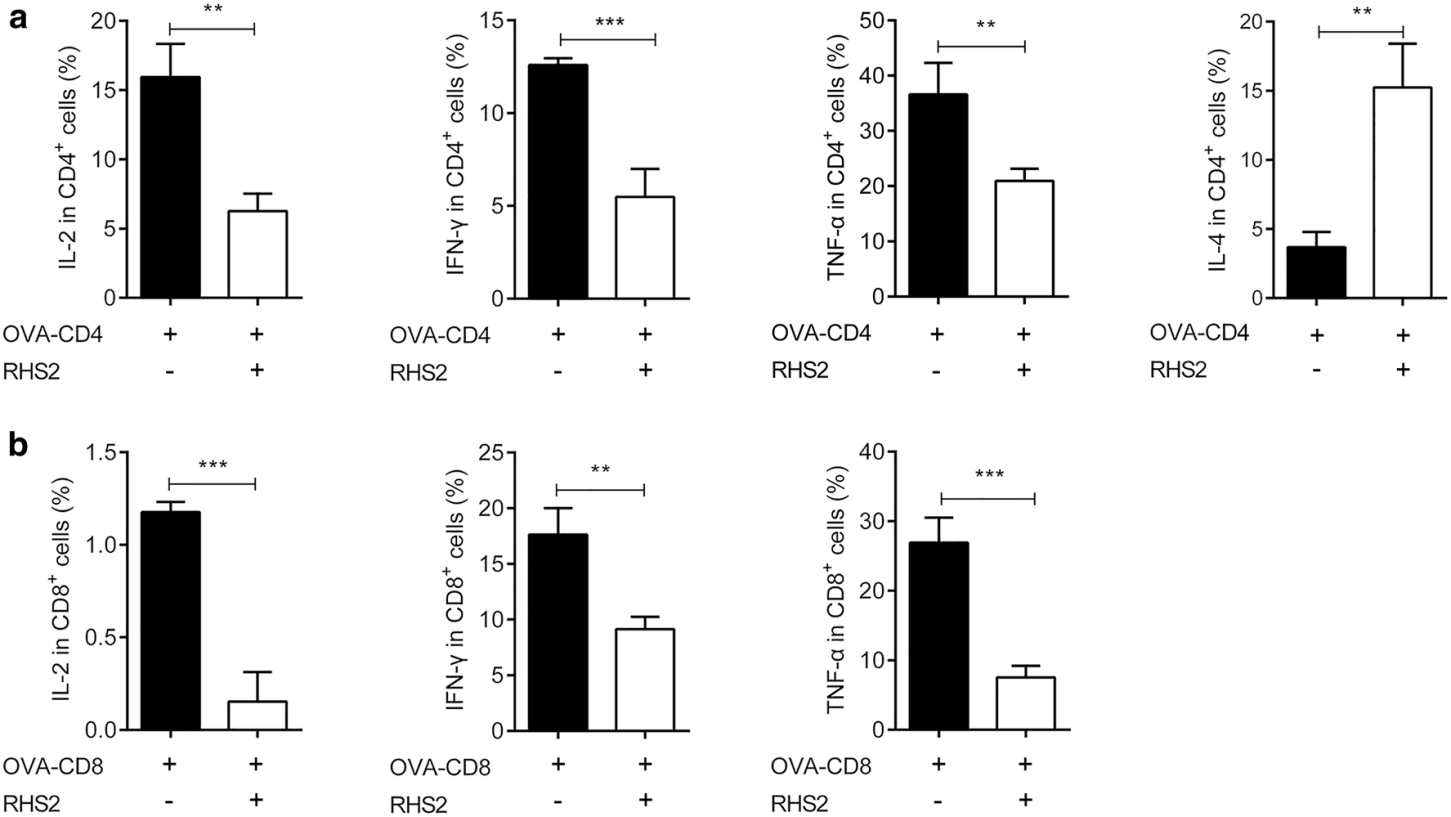
Production of IL-2, IFN-γ and TNF-α-from CD4^+^ and CD8^+^ T cells. **a** Co-cultured cells stained with anti-CD3^+^ and CD4^+^ markers were analyzed by flow cytometry to evaluate levels of IL-2, IL-4, IFN-γ and TNF-α. The percentage of IL-2-, IL-4-, IFN-γ- and TNF-α-producing cells was determined in live CD4^+^ cells. **b** Co-cultured cells stained with anti- CD3^+^ and CD8^+^ markers were analyzed by flow cytometry to evaluate levels of IL-2, IFN-γ and TNF-α. The percentage of IL-2-, IFN-γ- and TNF-α-producing cells was determined in live CD8^+^ cells. The data are expressed as the mean percentages of production cytokines in CD4^+^ and CD8^+^ cells from triplicate wells plus SEM. *P < 0.05, **P < 0.01, ***P < 0.001

Since T cell activation by vaccine-primed DCs is important for adaptive immunity [50], we investigated the ability of OVA-CD4 polypeptide or OVA-CD8 polypeptide with RHS2-primed BMDC to activate T cells. We stimulated BMDC with OVA-CD4 or OVA-CD8 and RHS2 for 36 h and co-cultured them with isolated spleen lymphocytes for 3 days. As shown in Fig. 4a, b, RHS2-primed BMDC remarkably inhibited the secretion of IL-2, IFN-γ and TNF-α by CD4^+^ (t-test: *t*_(4)_ = 6.204, *P* = 0.0034; *t*_(4)_ = 7.934, *P* = 0.0006; *t*_(4)_ = 4.446, *P* = 0.005) and CD8^+^ (t-test: *t*_(4)_ = 10.45, *P* = 0.0005; *t*_(4)_ = 5.624, *P* = 0.0049; *t*_(4)_ = 8.425, *P* = 0.0005) cells. However, a significant increment was detectable following RHS2 treatment for IL-4 (*t*_(4)_ = 7.213, *P* = 0.002) after OVA-CD4 peptide stimulation (Fig. 4a). These results suggested that RHS2 stimulated DCs to inhibit Th1 immune responses in CD4^+^ T cells.

### Inhibition of OVA-CD8 induce cytotoxic T lymphocytes

**Fig. 5:**
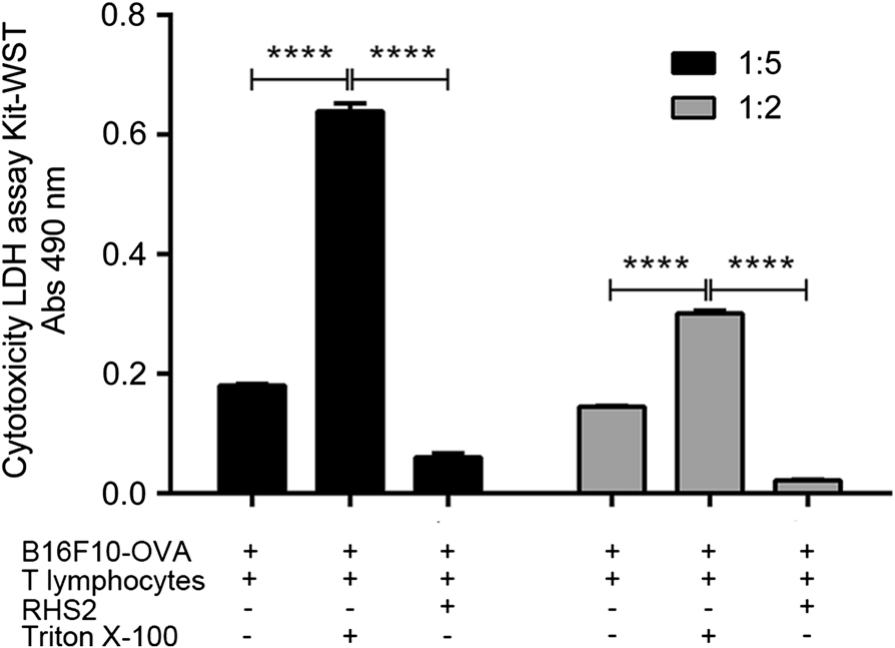
Cytotoxicity assay. OVA-CD8-activated CD8^+^ cytotoxic lymphocytes were used as effector cells, whereas the OVA-expressing B16F10 cells were used as target cells. Triton X-100 was added to the positive control group. The data are presented as the percent specific lysis of the target cells in a 2 h RHS2-stimulate assay. Each point represents the mean of triplicate cultures. *P < 0.05, **P < 0.01, ***P < 0.001, ****P < 0.0001

To evaluate the specific cytotoxic activity of CD8^+^ T lymphocytes generated after the immunization of C57BL/6 mice with the OVA-CD8 peptide, we cultured spleen lymphocytes cells from OVA immunized mice with OVA-CD8 for 4 days. Then, we analyzed the lytic activity of preculture B16F10-OVA cells and spleen lymphocytes at ratios of 1:2 and 1:5, and in the presence or absence of 20 μg/ml RHS2 protein. The results that are shown in Fig. 5 indicate that CD8^+^ T lymphocytes incubated with the OVA-CD8 peptide were able to lyse the B16F10-OVA cells, while spleen lymphocytes cells incubated with RHS2 protein showed a significantly lower lytic activity (one-way ANOVA: *F*_(2, 6)_ = 1206, *P* = 0.00001).

### Serum IgG1, IgG2a, IgG2b, IgM and IgE Level

**Fig. 6:**
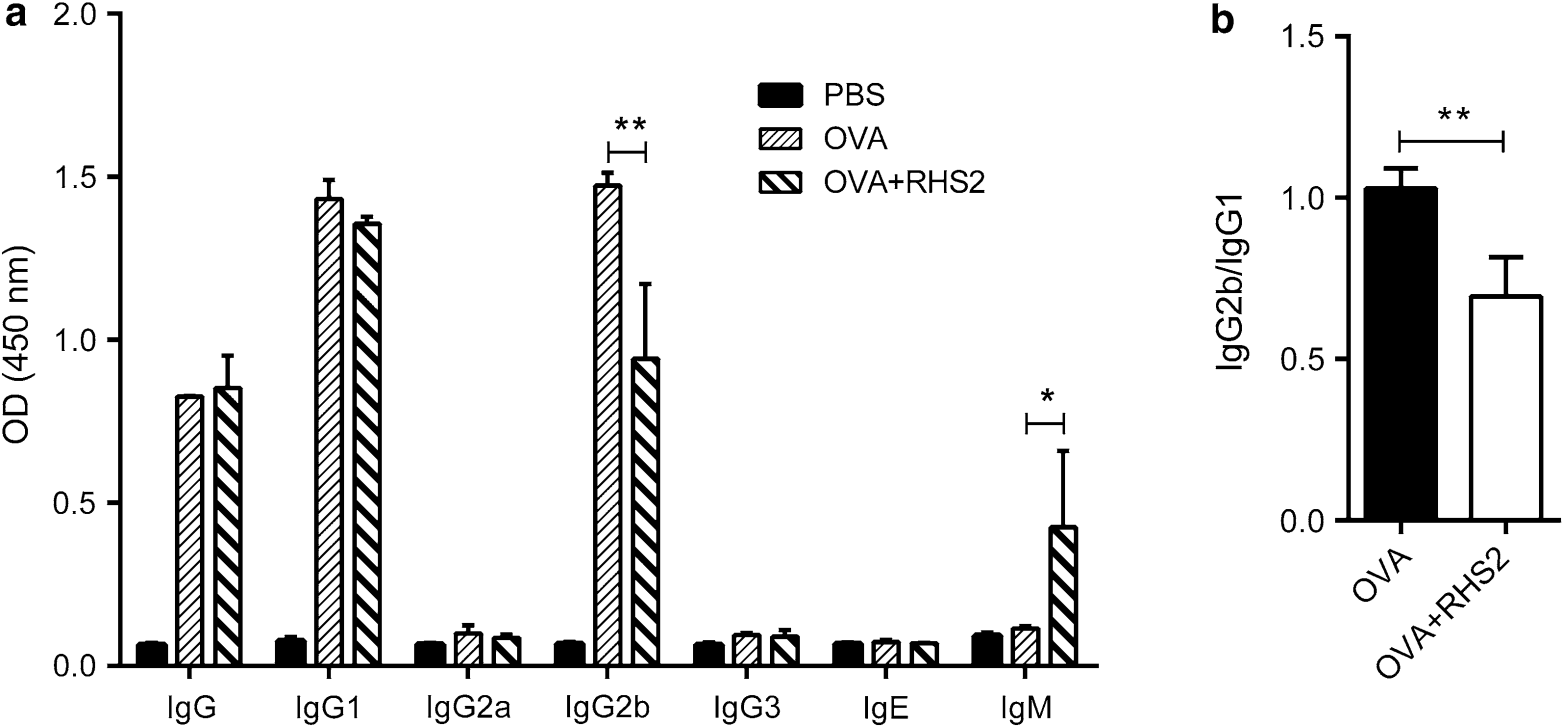
Effect of RHS2 on serum anti-OVA antibody in mice immunized subcutaneously. The serum was collected 10 days after the third immunization for antibody subtype and total antibody titer evaluation. **a** Anti-OVA antibody IgG, IgG1, IgG2a, IgG2b, IgG3, IgM and IgE were measured by ELISA. **b** IgG2b/IgG1 ratio. Each data point represents the mean antibody titer ± SEM with *n* = 5. *P < 0.05, **P < 0.01

Serum antibody levels of IgG1, IgG2a, IgG2b, IgG3, IgM and IgE induced by OVA were determined by ELISA. As expected the total IgG, IgG1 and IgG2b levels in the serum were significantly higher in the OVA group compared with the PBS group (one-way ANOVA: *F*_(2, 6)_ = 181.2, *P* = 0.00001; *F*_(2, 6)_ = 1315, *P* = 0.00001; *F*_(2, 6)_ = 163, *P* = 0.00001), whereas IgG2b from the OVA+RHS2 group was significantly lower compared with the OVA group (*F*_(2, 6)_ = 163, *P* = 0.001; Fig. 6a). There were significant differences in IgM (*F*_(2, 6)_ = 10.56, *P* = 0.0108) production for the OVA+RHS2 group relative to the control group (Fig. 6a), but there were no significant differences in IgG2a (*F*_(2, 6)_ = 5.879, *P* = 0.2339), IgG3 (*F*_(2, 6)_ = 5.041, *P* = 0.0519) and IgE (*F*_(2, 6)_ = 2.562, *P* = 0.1569) production.

Th1 cells induce the production of IgG2a and IgG2b antibodies, and Th2 cells promote IgE and IgG1 subclasses [51, 52]. IgG2b can be produced in response to either Th1 or Th2 under different conditions [53]. IgG2b and IgG1 are produced *via* Th1 and Th2 cells, which drive cellular and humoral immunity, respectively. The decreased ratio of IgG2b/IgG1 compared to the antigen control group is an indicator of Th1-biased immune response. As shown in Fig. 6b, antigens from the OVA+RHS2-treated mice exhibited a lower IgG2b/IgG1 ratio than the mice in the control group (t-test: *t*_(4)_ = 4.278, *P* = 0.0064; Fig. 6b).

## Discussion

Once on the host, ticks actively search for a favorable place for feeding. The first step in feeding is the secretion of a substance that solidifies the contact with the skin of the host. Adult females of the genus *Ixodes* imbibe blood meals exceeding about 100 times their own weight within 7–9 days. The success rate of pathogens transmitted by ticks is mainly a result of the favorable aspects of tick physiology arising from their adaptation to the relatively long-lasting blood-feeding [12]. During the progress of blood-sucking, a dramatic series of changes occurs in the tick’s salivary glands, whose cells are profoundly transformed, adapting their physiology and pharmacological secretions to the new ‘active’ status [54]. The inoculation of several dozen pharmacologically active compounds [54–56] contributes to the sustained flow of blood into the feeding cavity, lysis of the cells surrounding the feeding place and evasion of the host’s immune response [12]. Ticks lack extracellular digestive enzyme organs, and ticks uptake components of host blood by endocytic digestive cells that line the tick midgut epithelium [16]. This is beneficial to the long-term survival of pathogens that are ingested into the midgut. Nevertheless, ticks have a defense mechanism that enables them to maintain pathogens and commensal microbes at a certain level [57], without compromising their own health and development. The long-term co-evolution of ticks and pathogens leads to mutual tolerance and adaptation of their physiological differences. Information on tick innate immunity is rather fragmented and allows only an approximate comparison with other invertebrates [58, 59]. Humoral defenses are based on a variety of pattern-recognition proteins and effector molecules, such as lectins, complement-related molecules and a broad spectrum of common as well as specific antimicrobial peptides (AMPs) [58].

Since ticks are blood-sucking arthropods, host immune responses are evaded by various mechanisms during blood-sucking. Many molecules in the saliva secreted into the biting site act on the host immune system, including anti-inflammatory molecules, protease inhibitors and innate and acquired immunomodulatory molecules. Serpin has a variety of biological functions, and it is secreted into the host by saliva during blood-sucking, acting as an anticoagulant [11, 21–24], immunosuppressant [14, 27] and enzyme inhibitory factor [11, 23, 60], which are beneficial to blood-sucking development and pathogen transmission.

In haematophagous ectoparasites, DC modulators have presumably evolved to suppress host immunity in order to facilitate blood-feeding [61]. The present study demonstrated that RHS2 from *R. haemaphysaloides* ticks was able to inhibit the differentiation and terminal maturation of DCs, and subsequently affected their immunostimulatory functions. This could be the mechanism by which blood-feeding arthropods immunosuppress their host’s immune response during meal uptake [62, 63], and it could also explain how tick saliva can modulate the production of cytokines by the host. The present investigation also showed what seems to be a novel modulatory role of arthropod vectors’ saliva at an initial step of the immune response through the inhibition of differentiation and maturation of DCs into functional antigen-presenting cells, which has also been supported by a previous report [26].

We found that tick RHS2 inhibited GM-CSF/IL-4-driven differentiation of BM precursors into DCs. The culture system using GM-CSF/IL-4 to differentiate BM precursors into DCs favors the expression of genes that are related to the development of these cells to the detriment of other haemopoietic populations, such as granulocytes and macrophages. DCs develop from progressively restricted bone marrow progenitors: this progenitor cells include granulocyte, monocyte and DC progenitor [64]. The diminished percentage of DCs found when RHS2 was present in the culture was not caused by cell death and was not compensated by B cells (CD19), but it was compensated by granulocyte (Gr-1^+^) and macrophage (F4/80^+^) cell populations.

To our knowledge, this study provides the first direct evidence that the JAK/STAT pathway results in the immune suppression of BMDC induced by tick serpin RHS2. Therefore, we conducted Western blot analysis to identify the signaling molecules involved. We examined the phosphorylation of ERK, p38 and STAT3, which are essential in regulating many cellular processes including inflammation, cell differentiation and cell proliferation. We also showed that tick serpin RHS2 could lead to BMDC immunosuppression. RHS2 could induce high expressions of p-STAT3 and p-p38, consequently regulating the surface marker, which might influence BMDCs to drive T helper 1 type immune responses. Furthermore, STAT3-depleted DCs are more resistant to cancer cell-derived inhibitory factors [65]. These STAT3-deficient DCs are more potent activators of T cells and they have a high capacity to induce Th1 responses [65]. Activation of p38 kinase is related to stress response, growth arrest and apoptosis [66, 67], whereas ERK is important in cellular mitogenesis and differentiation [68]. MAPK activation regulates gene expression *via* the phosphorylation of downstream transcription factors.

Upon exposure to inflammatory or microbial stimuli such as LPS or TNF-α, immature DCs upregulate the surface expression of MHCII, beside adhesion and co-stimulatory molecules [36, 37]. After stimulation, these newly matured DCs reduce their antigen-uptake capacity and migrate to the regional lymph nodes, where they exert their function as potent APC [37]. The results presented herein demonstrated that RHS2 could inhibit the expression of CD80, CD86 and MHCII induced by LPS on the DCs’ surfaces. However, in this study, the expression of CD40 molecules was not altered. Fewer CD40 molecules on the DCs can result in diminished presentation of antigen, reduced production of cytokines and, as a consequence, lack of CD80 and CD86 upregulation. Our results showed that RHS2 may impair the competence of DCs for T-cell priming, owing to a lack of appropriate co-stimulation.

The flow cytometry data showed that RHS2 could result in the downregulation of CD80, CD86 and MHCII expression. The antigen-presenting function of DCs is dependent on their activation, which is characterized by the expression of MHCII molecules and co-stimulatory molecules such as CD80. It has been reported that the low level of MHCII could reduce DCs’ production of inflammatory cytokines in response to Toll-like receptor ligands. It also weakened DCs ability to interfere with antigen-specific CD4^+^ T cell activity in regulatory T-cell development [69]. Reduced CD80 expression correlates with reduced IFN-γ levels by increasing the Th2 cytokine levels [70] and the suppression of the Th1 response [71] in reflecting the negative influence of antigens on DCs. Here, we found that the lower level expression of CD80, CD86 and MHCII resulted in poor CD4^+^ and CD8^+^ T-cell stimulatory capacity. A decreased number of CD4^+^ and CD8^+^ T cells might be implicated in autoimmune and inflammatory disorders; many chronic diseases, including cancer, are linked to inflammation disorder [72]. The balance between the different immunological cells is responsible for a normal immunological function to maintain immune homeostasis.

This study showed that RHS2 significantly influenced the equilibrium between Th1 and Th2 subpopulations and the cytokines they produce. Each subpopulation is characterized by unique transcription factors and cytokine patterns. CD4^+^ Th lymphocytes can be divided into Th1 (IFN-γ, IL-2 and TNF-α) and Th2 (IL-4, IL-5, IL-6, IL-10 and IL-13) subsets on the basis of their cytokine secretion profile [73–75]. Th1 cytokines induce the cell-mediated immune response targeted against intracellular pathogens [76]. They promote isotype class switching to produce IgG2 [73, 77] and they trigger antibody-dependent cellular cytotoxicity. Th2 cytokines induce powerful antibody-mediated responses [78]. Antibody isotypes switch to IgG1 [73] and IgE, which protects the body against extracellular pathogens, in particular parasites. Th1 and Th2 cytokines are produced by T cells and other immune system cells and changes in Th1/Th2 cell polarization of an immune response are associated with susceptibility to autoimmune and infectious diseases.

In addition, CD8^+^ T cells perform a cytotoxic function *via* the quality of antigen presentation [79]. The number of CD3^+^CD8^+^ T cells secreting IFN-γ, IL-2 and TNF-α was decreased, indicating that CD8 T cell activation was inhibited. It is well established that CD8^+^ T cells constitute an important branch of adaptive immunity that contributes to clearance of intracellular pathogens and provides long-term protection. These functions are mostly fulfilled by the best characterized subpopulation of CD8^+^ T cells, the cytotoxic T lymphocytes, owing to their ability to kill infected cells and to secrete cytokines such as IFN-γ and TNF-α [80]. However, our results indicated that RHS2 inhibited the killing of target cells by cytotoxic T lymphocytes. However, it is not clear that RHS2 inhibits CD8^+^ T cells from killing cells bearing the target antigen by inhibiting the release of cytotoxic molecules (such as granzymes and perforin), or inhibiting the secretion of cytokines (IFN-γ and TNF-α) into the immunological synapse.

According to the secreted cytokine profile, differentiation of naïve CD4^+^ cells into functionally distinct effector helper T cell subsets [81–83], with Th1 and Th2 populations, is the most thoroughly studied in the tick-host interaction. Th1 populations are associated with host cellular and inflammatory responses and Th2 populations are linked with host humoral responses against ticks [74, 75].

Experiments with tick serpin have shown polarization of the immune response from Th1 to Th2 branches by suppression of Th1 and upregulation of Th2 cytokines in both mice and humans. This polarization leads to an attenuated inflammatory response, which is beneficial for tick survival and feeding [84]. Briefly, RHS2 inhibited secretion of IL-2, TNF and IFN-γ. One of the mechanisms described for the action of *R. haemaphysaloides* serpin involves a negative effect on DCs, which then prime naive CD4^+^ T cells to induce Th2 cell differentiation *in vitro* and *in vivo*. Ticks have evolved various methods to circumvent adaptive immunity and to actively direct the immune response toward the Th2 arm, which favors their feeding. The immunosuppressive properties of tick secretions also include the inhibition of antibody production by B cells, which could damage activation of other cells or complement.

## Conclusions

To our knowledge, this is the first report demonstrating that RHS2 has an inhibitory effect on the initial, activation and effect phases of the host immune response. RHS2 from *R. haemaphysaloides* ticks was able to inhibit the differentiation and terminal maturation of DCs, subsequently affecting their immunostimulatory functions. RHS2-primed BMDC remarkably inhibited the secretion of IL-2, IFN-γ and TNF-α by CD4^+^ and CD8^+^ cells, as well as Th1 immune responses. Ticks have evolved various ways to circumvent adaptive immunity, which is beneficial for their survival and feeding, and the transmission of TBDs.

## Data Availability

All data generated or analyzed during this study are included in this article.

## Abbreviations

serpin: serine protease inhibitor
RHS2: *R. haemaphysaloides* serine protease inhibitor 2
CTLs: cytotoxic T lymphocytes
BMDC: bone marrow-derived dendritic cells
DCs: dendritic cells
OVA: ovalbumin
TBD: tick-borne diseases
IL-2: interleukin 2
IL-4: interleukin 4
IFN-γ: interferon gamma
TNF-α: tumor necrosis factor alpha
MHCI: major histocompatibility complex I
MHCII: major histocompatibility complex II
LPS: lipopolysaccharide
IPTG: isopropyl β-D-1-thiogalactopyranoside
GM-CSF: granulocyte-macrophage colony stimulating factor
SDS-PAGE: sodium dodecyl sulfate-polyacrylamide gel electrophoresis
FBS: fetal bovine serum
PBS: phosphate-buffered saline
PMSF: phenylmethylsulfonyl fluoride
TBS: Tris-buffered saline
SMP: skimmed milk powder
ELISA: enzyme-linked immunosorbent assay
